# Evaluation of nitazene immunoassay test strips for rapid in-situ detection of nitazene and nitazene analogs in illicit drug samples

**DOI:** 10.1186/s12954-025-01287-9

**Published:** 2025-08-09

**Authors:** Victoria Marland, Lorna Nisbet, Niamh Nic Daéid

**Affiliations:** Leverhulme Research Centre for Forensic Science, School of Science and Engineering, University of Dundee, Dundee, UK

**Keywords:** Drug checking, Nitazene, Immunoassay test strips, Harm reduction, Novel synthetic opioids

## Abstract

**Background:**

The detection of nitazene compounds in the United Kingdom has raised concerns among healthcare professionals, public health authorities, and law enforcement due to the increased risk of fatal overdose, particularly among opioid users. In response, nitazene testing strips have been distributed to help users identify these substances in drugs they may consume. However, to date, limited testing has been conducted on the effectiveness of these strips.

**Methods:**

This study assesses the sensitivity and selectivity of a widely distributed nitazene immunoassay drug testing strip. The limit of detection and selectivity was examined for 36 nitazene analogs and 93 other drugs and cutting agents commonly encountered in illicit samples. The effectiveness of the test strips for the detection of metonitazene in the presence of other drugs was examined using a series of concentrations in solution in combination with other drugs. Testing of the strips was also carried out using authentic seized heroin samples.

**Results:**

The strips detected 28 out of 36 nitazene compounds (78%) with detection limits ranging from 250 ng/mL to 100 µg/mL. The strips did not provide positive results for 93 other drugs and cutting agents at a concentration of 100 µg/mL. However, false positives were observed when testing seized heroin samples, caused by caffeine concentrations over 300 µg/mL. False negatives were also seen for eight nitazene compounds.

**Conclusion:**

Caution should be exercised when deploying these nitazene test strips as frontline presumptive tests in both criminal justice and public health contexts due to the observed false negative and false positive results. While the strips successfully detected a majority of nitazene compounds, their inability to identify certain analogs poses a risk of not detecting the presence of these potent opioids in the drug supply. Additionally, the occurrence of false positives, due to the presence of caffeine—a common adulterant in illicit substances—raises concerns about their suitability for use as a harm reduction method.

**Supplementary Information:**

The online version contains supplementary material available at 10.1186/s12954-025-01287-9.

## Introduction

Drug related deaths (DRDs) in Scotland are the highest within the United Kingdom and Europe with a total of 1197 suspected drug related deaths occurring in 2023 [1]. These figures represent a 12% increase on DRDs since 2022, driven by a 16% increase in males specifically. The majority of DRDs in Scotland involve opiates, implicated in 80% of DRDs in 2023, followed by benzodiazepines present in 58% of DRDs.

To date, opiate drug related deaths within the United Kingdom and Scotland, have not been as heavily impacted by the rise of synthetic opioids such as fentanyl and their analogues, as has been evidenced in other countries including the United States [2, 3]. For example, in Scotland, there has only been a minor increase in fentanyl detections reported to Public Health Scotland in post-mortem toxicology data, from 1% in Q1 of 2022 to 8% in Q4 of 2024. Despite this, there are growing concerns around the increasing number of detections of 2-benzyl benzimidazole ('nitazene') opioids which were detected in Scottish DRDs for the first time in 2022 (1 DRD, 0.1%) and in 31 (2.6%) DRDs in Scotland in 2023 [3–5].

Nitazenes were developed in the 1950s as opioid analgesics but failed to receive medicinal approval [6]. The potency of these compounds varies significantly between analogues [7]. Nitazene compounds are frequently found in combination with other opioids such as diamorphine as part of heroin mixtures and as such it is unlikely that users will have any knowledge that they are administering a nitazene containing substance, much less the specific nitazene in question [8]. Potentially more concerning is the detection of nitazenes in non-opiate mixtures, posing an added risk of these substances being unwillingly ingested by opiate naïve users [9].

Concerns around the dangers associated with nitazene compounds, and the increase in nitazene involvement in DRDs worldwide resulted, in isotonitazene, metonitazene, protonitazene, etonitazepyne and etazene being placed under international control by the United National Office for Drug Control (UNODC) [10]. Further nitazene analogues are scheduled to be added to this list.

The use of commercially available testing strips has been previously investigated as a harm reduction strategy, most recently to address the DRDs associated with fentanyl in the USA and Canada [11–14]. The impact of drug testing strips upon user behavior is mixed, with research suggesting that factors such as where drugs are to be consumed and the perception of drug quality playing a pivotal role [11–14]. Previous research has found that providing testing strips resulted in users changing their drug use behavior, including consuming substances in the presence of another individual, reducing the amount consumed and, in some cases, discarding the drug completely. Drug testing strips are also appealing to those working within harm reduction services as they are simple to undertake, non-invasive, portable and cheap to purchase, requiring no prior chemistry knowledge [11–14].

When a new technique, such as an immunoassay testing strip, is used within a laboratory setting, it must undergo a range of quality assurance testing before being deemed effective. This involves assessing the cut-off concentration or limit of detection, the point at which the strip no longer detects the compound(s) for which it has been designed, and its cross-reactivity: whether the strip produces a positive response in the presence of other substances [15]. Although the majority of commercially available products will be accompanied by leaflets providing this information, this should always be validated prior to use within justice or public health settings.

The validation of drug testing strips prior to distribution poses inherent challenges for those in the harm reduction sector, as it involves handling controlled substances at known concentrations. Conducting this work is typically limited to those with government authorization, typically in the form of a license allowing them to purchase, use, and store these substances. Consequently, many of these strips are distributed without verifying the accuracy of the claims stated in the product inserts. There are concerns that lack of validation could reduce the trust and credibility of services if strips are found to cross react with other commonly encountered substances.

In this study we evaluated a commercially available Rapid Response™ Nitazene Test Strip manufactured by BTNX for their ability to detect isotonitazene. We also assess the ability of these strips to detect additional nitazenes as well as their effectiveness in the presence of other commonly encountered licit and illicit drugs of abuse and cutting agents. Finally, we assessed the strips using authentic seized heroin samples.

## Materials and methods

### Materials

Rapid Response™ Nitazene Test Strips (Lot numbers DOAB24050015 and DOAB24020005) manufactured by BTNX (Ontario, Canada) were purchased from Exchange Supplies (Dorset, UK). Methanol (MeOH) and acetonitrile (ACN) were purchased from Fischer Scientific (Leicestershire, UK). Tap water, from the laboratory, was used in place of deionized water to better reflect real-world conditions under which the samples are likely to be prepared, as end-users of the product may not have access to deionized water. The reference standards used in this study are listed in Supplementary 1. Authentic seized samples were provided by the Scottish Prison Service (SPS).

### Methods

#### Sampling method using test strips

The manufacturer instructions for the Rapid Response™ Nitazene Test Strips reported the limit of detection for isotonitazene, protonitazene and *N*-pyrrolidino etonitazene of 2000, 3000 and 1300 ng/mL respectively.

**Fig. 1:**
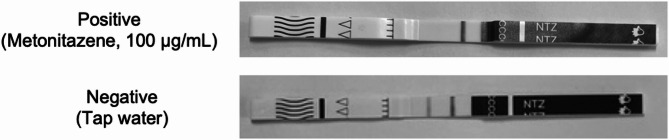
Examples of positive and negative test strip results

The test strips were used according to the manufacturer’s instructions. Each test strip was removed from the individual sealed pouch and dipped into a prepared solution containing the target drug at a known concentration, for approximately 10–15 s. The test strip was placed on a flat dry surface and the result was read within the manufacturer’s recommended time limit of 10 min, although results were typically available and recorded after 1 min. Test strips were recorded as positive if one line was visible on the test strip, and negative where two lines were visible. Strips which failed to produce any lines, or no control line, were marked as invalid and repeated. Examples of a positive test strip (metonitazene) and a negative test strip (tap water) can be seen in Fig. 1.

#### Nitazene selectivity and cross reactivity

Solutions of 100 µg/mL of each nitazene, drug or cutting agent were prepared, using certified reference standards, in tap water. Where the certified reference standard was a neat powder, 1 mg was dissolved into 1 mL of tap water, to produce a 1 mg/mL solution, and diluted using tap water to achieve a 100 µg/mL solution. Where the certified reference standard was a 1 mg/mL solution, 100 µL was added to 900 µL of tap water to achieve a 100 µg/mL solution. The nitazene test strips were then used to sample these solutions as described in Section “Sampling method using test strips”. A negative control was also carried out containing only tap water to ensure that any response was due to the presence of the nitazene.

##### Limit of detection (LOD)

Solutions of 2000 ng/mL in tap water were prepared for each nitazene that provided a positive result during cross reactivity testing. This initial concentration was based on the manufacturer's instructions which stated that the limit of detection of the test strips for isotonitazene was 2000 ng/mL. This solution was tested in triplicate and if the test strips produced a positive result, the solution was diluted using a 1 in 2 dilution until the test-strips provided a negative result. In the event that an analog produced a negative result in the 2000 ng/mL solution, a fresh solution at a higher concentration was tested. This process was repeated until the test strip produced a positive result. Each concentration was analyzed in triplicate. This study defined the limit of detection (LOD) as the lowest concentration at which all replicates produced a positive result.

##### Nitazene detection in mixtures

Metonitazene was selected to evaluate the detection of nitazenes in the presence of other compounds. This analog was chosen as it was the most commonly reported nitazene analog in the UK, at time of testing, in samples submitted to the Welsh Emerging Drugs and Identification of Novel Substances Project (WEDINOS) [9]. Solutions of 1300 ng/mL metonitazene and 100 µg/mL of the other drugs or cutting agents (Table 1) were prepared in tap water and tested using the test strips according to the manufacturer’s instructions. Drugs and cutting agents were selected based on those mostly commonly detected within non-judicial samples from Scottish Prisons and those mostly commonly detected in mixtures with nitazenes as reported in samples submitted to the Welsh Emerging Drugs and Identification of Novel Substances Project (WEDINOS) [9].

**Table 1:** List of compounds used for evaluating the detection of metonitazene in mixtures

Compound (100 µg/mL)
ADB-BUTINACA
Benzocaine
Bromazolam
Brorphine
Caffeine (Anhydrous)
Cocaine (Base)
Cocaine hydrochloride
Codeine hydrochloride
Diamorphine (base)
Diazepam
Dimethylpentylone hydrochloride
Etizolam
Gabapentin
Glucose
Ketamine hydrochloride
Levamisole hydrochloride
Lignocaine
Magnesium sulphate
MDMA hydrochloride
MDMB-4en-PINACA
Methadone hydrochloride
Methamphetamine hydrochloride
Methandienone
Oxymetholone
Paracetamol
Phenacetin
Sodium bicarbonate
Stanozolol
Xylazine

##### Analysis of seized heroin samples

Three seized heroin samples, obtained from Scottish prisons were used to assess the strips performance using authentic samples. Approximately 1 mg of each seized sample was dissolved in 1 mL of tap water, and vortexed for approximately 30 s, until fully dissolved, to produce a 1 mg/mL solution. The nitazene test strips were then used to sample these solutions as described in Section “Sampling method using test strips”. A negative control was also carried out containing only tap water.

## Results

### Nitazene selectivity and limit of detection (LOD)

The nitazene test strips delivered positive detections for 28 of the 36 nitazenes tested. The test strips failed to positively identify 3-methoxymetodesnitazene, 5-methyl etazene citrate, etazene, ethylene etonitazene, metodesnitazene, protodesnitazene, *N*-pyrrolidino metodesnitazene and 5-aminoisotonitazene as shown in Table 2. The chemical structure of each compound listed in Table 2 can be found in Supplementary 2.

**Table 2:** Selectivity and limit of detection when using the nitazene test strips as identified through this research

Drug	Result at 100 µg/mL	Limit of detection (ng/mL)
Metonitazene hydrochloride	Positive	1000
Etonitazene	Positive	1000
Protonitazene hydrochloride	Positive	3000
Butonitazene	Positive	5000
Isotonitazene	Positive	1500
Menitazene citrate	Positive	2000
Clonitazene	Positive	3000
Flunitazene hydrochloride	Positive	2000
Propylnitazene citrate	Positive	2000
Sec-butonitazene	Positive	2000
Methylenedioxy nitazene citrate	Positive	2000
Ethylene oxynitazene citrate	Positive	2000
Ethylene etonitazene citrate	Negative	N/A
Metodesnitazene hydrochloride	Negative	N/A
Etazene hydrochloride	Negative	N/A
Protodesnitazene	Negative	N/A
5-Methyl etazene citrate	Negative	N/A
5-Methyl metodesnitazene citrate	Positive	100,000
3-Methoxy metodesnitazene citrate	Negative	N/A
Fluetonitazene citrate	Positive	1000
4′-hydroxynitazene	Positive	1000
*N*-desethyl metonitazane hydrochloride	Positive	250
*N*-desethyl etonitazene	Positive	500
*N*-desethyl protonitazene hydrochloride	Positive	2000
*N*-desethyl isotonitazene hydrochloride	Positive	3000
*N*-piperidinyl 4'-hydroxynitazene citrate	Positive	500
*N*-piperidinyl metonitazene citrate	Positive	1000
*N*-piperidinyl protonitazene citrate	Positive	3000
*N*-piperidinyl isotonitazene citrate	Positive	2000
*N*-pyrrolidino etonitazene	Positive	1500
*N*-pyrrolidino isotonitazene citrate	Positive	1000
*N*-pyrrolidino fluetonitazene citrate	Positive	1000
*N*-pyrrolidino 4'-hydroxynitazene citrate	Positive	250
*N*-pyrrolidino metonitazene citrate	Positive	1000
*N*-pyrrolidino metodesnitazene citrate	Negative	N/A
5-Amino isotonitazene	Negative	N/A

As shown in Table 2, there was a wide variation in the LOD for each nitazene, ranging from 250 to 100,000 ng/mL. The manufacturer, BTNX, has only reported the LOD for three of the compounds included in this study; isotonitazene, protonitazene and *N*-pyrrolidino etonitazene, which are listed in the product insert as 2000, 3000 and 1300 ng/mL respectively. In contrast, the results of this study indicated LODs of 1500 ng/mL for isotonitazene, 3000 ng/mL for protonitazene HCl, and 1500 ng/mL for *N*-pyrrolidino etonitazene. These minor discrepancies may be attributed to differences in experimental conditions, sample matrices, or compound salt forms. Overall, the findings of this study support the manufacturer’s general sensitivity claims.

### Cross reactivity

The nitazene test strips were tested against 93 drugs and cutting agents including, but not limited to, 26 non-nitazene opioids, 30 synthetic cannabinoids and 15 benzodiazepines. A full list of these compounds, and the test results can be found in Supplementary 3. The nitazene test strips did not produce a positive result for any of these compounds at concentrations of 100 µg/mL (supplementary file  1).

### Nitazene mixtures

Solutions containing 1300 ng/mL of metonitazene hydrochloride were prepared containing 100,000 ng/mL of another drug as listed in Table 3. 1300 ng/mL was selected as a suitable concentration for metonitazene hydrochloride as this concentration is close to the LOD for the compound (1000 ng/mL) and mimics a scenario in which nitazenes are present as a minor constituent of a mixture. As shown in Table 3 a positive result was obtained for all solutions confirming that the test strips can detect metonitazene hydrochloride when present in combination with these compounds.

**Table 3:** Test strip results when used to test for the presence of metonitazene hydrochloride (1300 ng/mL) in mixtures

	Compound in mixture (100,000 ng/mL)	Test strip result
Metonitazene 1300 ng/mL	ADB-BUTINACA	Positive
	Benzocaine	Positive
	Bromazolam	Positive
	Brorphine	Positive
	Caffeine (anhydrous)	Positive
	Cocaine (base)	Positive
	Cocaine hydrochloride	Positive
	Codeine hydrochloride	Positive
	Diamorphine (base)	Positive
	Diazepam	Positive
	Dimethylpentylone hydrochloride	Positive
	Etizolam	Positive
	Gabapentin	Positive
	Glucose	Positive
	Ketamine hydrochloride	Positive
	Levamisole hydrochloride	Positive
	Lignocaine	Positive
	Magnesium sulphate	Positive
	MDMA hydrochloride	Positive
	MDMB-4en-PINACA	Positive
	Methadone hydrochloride	Positive
	Methamphetamine hydrochloride	Positive
	Methandienone	Positive
	Oxymetholone	Positive
	Paracetamol	Positive
	Phenacetin	Positive
	Sodium bicarbonate	Positive
	Stanozolol	Positive
	Xylazine	Positive

### Analysis of seized heroin samples

**Fig. 2:**
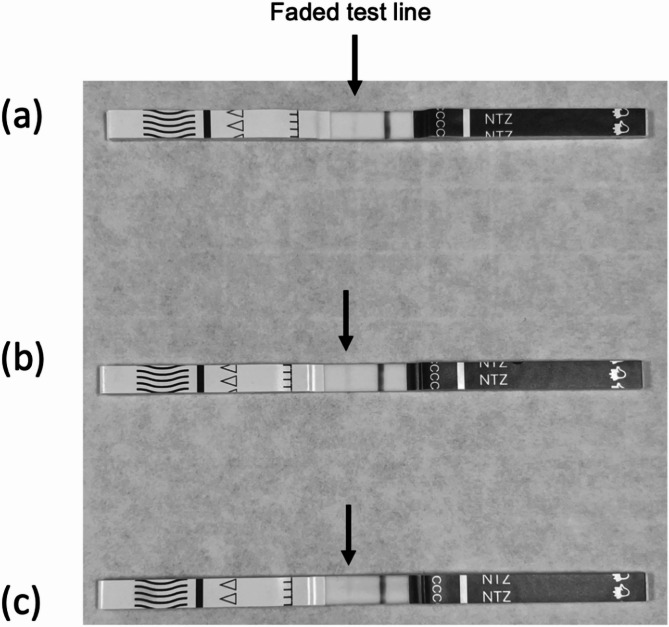
Images of testing strips following the analysis of 3 seized heroin samples from Scottish prisons. Each test trip displays a faded test line (indicated by black colored arrows), suggesting a degree of cross reactivity with the test strip and the seized samples

During this study, informal reports were received from harm reduction services and Public Health Scotland, that false positive results were being obtained when applying these test strips to heroin street samples. To investigate these reports the test strips were applied to three seized heroin samples from Scottish prisons. It should be noted that the testing guidelines provided by BTNX state that any presence of a line, even if faint, should be interpreted as a negative result. Each of the seized samples did present a faint line (Fig. 2) and by manufacturer’s guidelines were therefore providing a true negative result. However, the high degree of fading in these samples suggests interference from a compound within the seized heroin sample.

Street heroin is a complex mixture of opiates and cutting agents and may contain residues from the extraction process of opiates from the poppy plant (*Papaver Somniferum*) which involves various chemicals such as acetic anhydride. Each of the seized heroin samples, analyzed using GC–MS, were found to contain the opiates diamorphine, papaverine, acetylcodeine, 6-monoacetylmorphine (6-MAM), noscapine as well as caffeine and paracetamol. Analysis of the extracted ion chromatograms confirmed the absence of nitazenes in these samples [16]. Full analytical data relating to the GC–MS analysis of these seized samples can be found in Supplementary 4.

This study, and previous studies such as Sisco et al. (2024) found no cross reactivity of the test strips with diamorphine, papaverine, acetylcodeine, 6-monoacetylmorphine (6-MAM), noscapine, caffeine or paracetamol at 100,000 ng/mL. However previous studies have suggested that compounds can start to interfere with immunoassay test strips at higher concentrations [17, 18]. To assess this, 2 mg/mL solutions of diamorphine (base), papaverine hydrochloride, noscapine, 6-acetylcodeine, caffeine and paracetamol were analyzed using the test strips. Due to the availability of reference standard material, it was not possible to carry this out for 6-MAM.

**Fig. 3:**
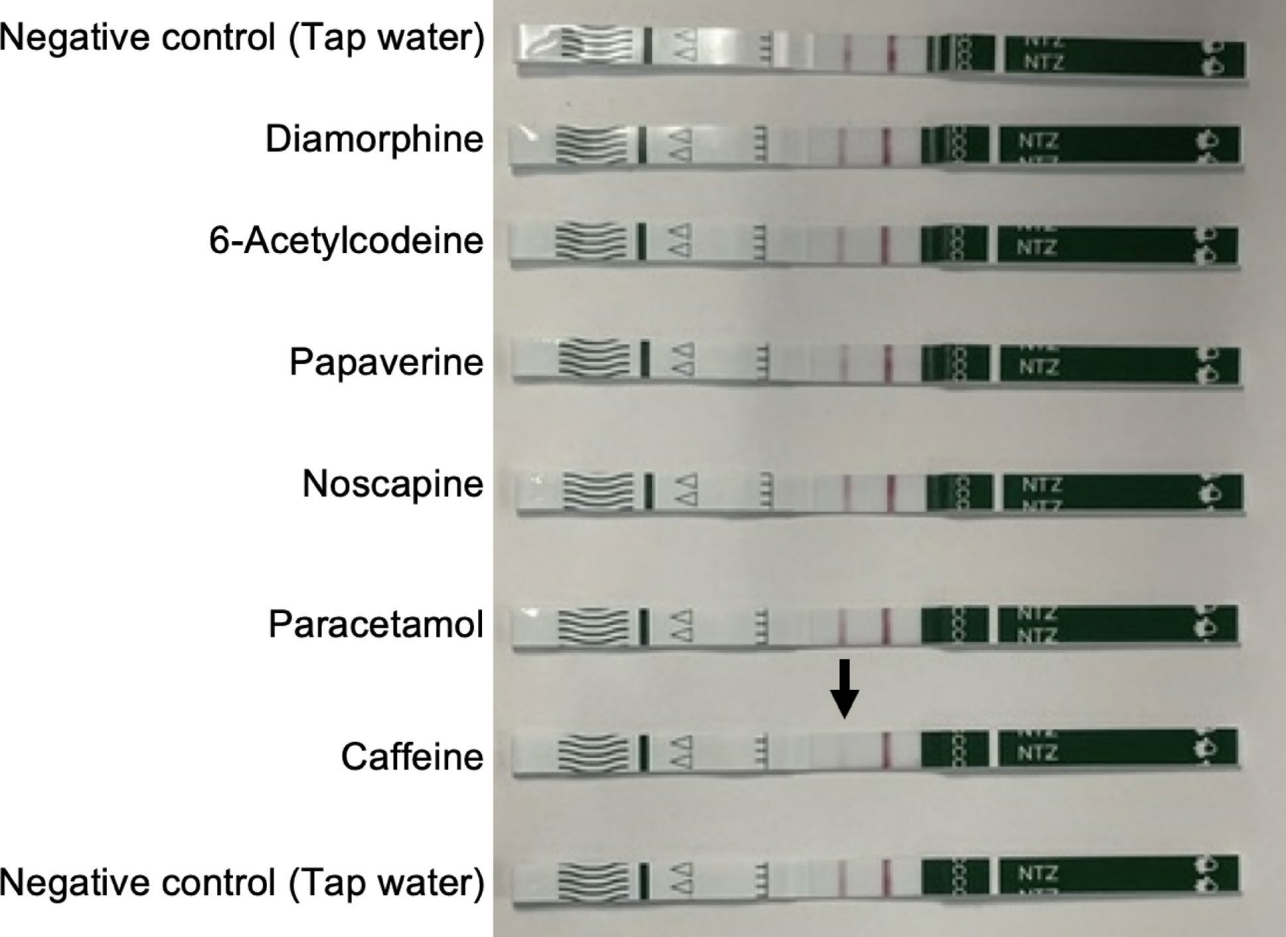
Images of testing strips following the analysis of 2 mg/mL solutions of diamorphine (base), 6-acetylcodeine hydrochloride, papaverine hydrochloride, noscapine (base), paracetamol and caffeine. The caffeine test strip displays a faded test line (indicated by black colored arrow), suggesting a degree of cross reactivity with the test strip and caffeine

No cross reactivity was observed with any of the opiate compounds or paracetamol. However, for caffeine a high degree of fading was observed, indicated by the black colored arrow in Fig. 3.

**Fig. 4:**
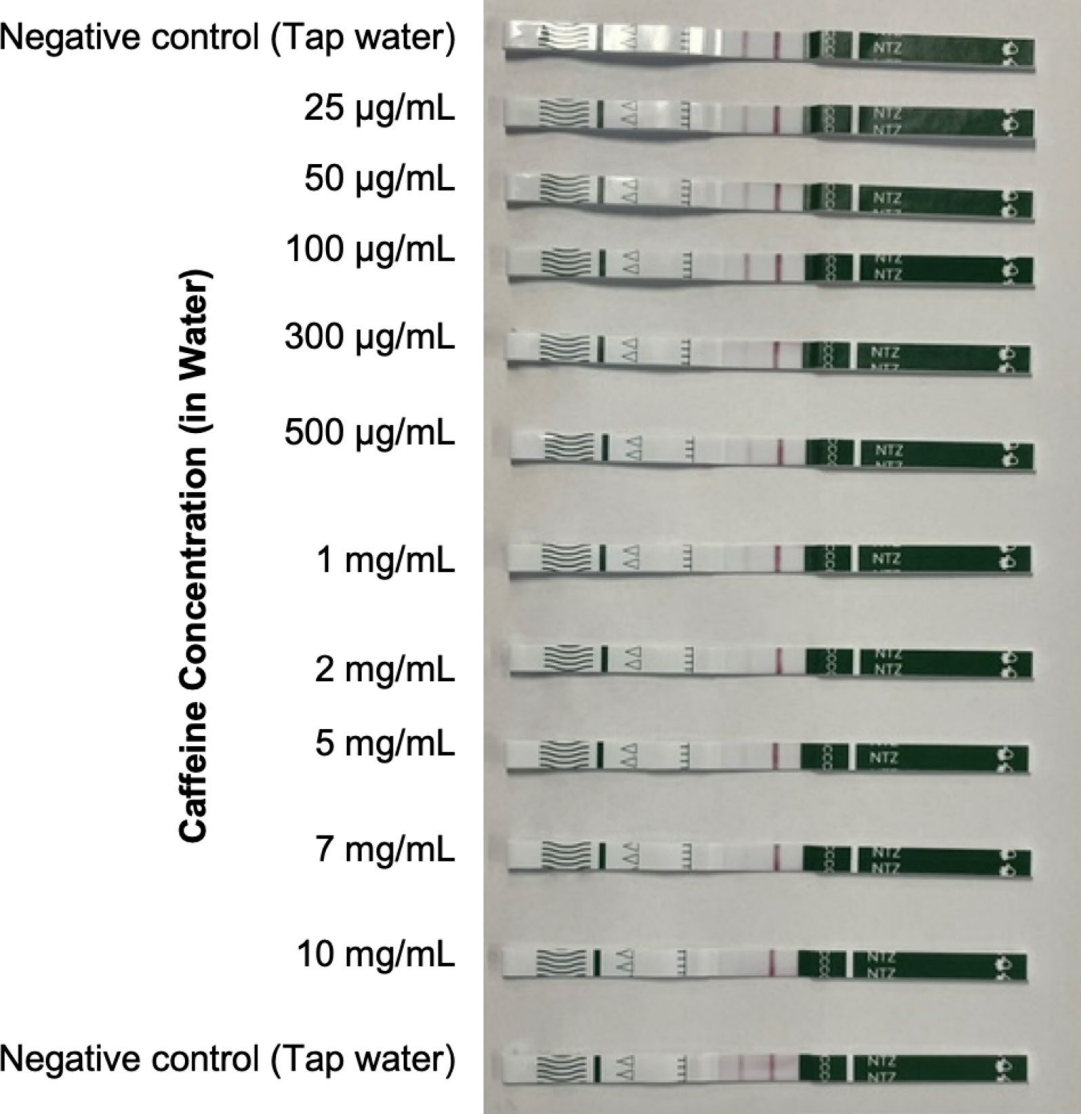
Images of testing strips following the analysis of a series of caffeine concentrations. The test line begins to fade at a concentration of 300 µg/mL

Earlier results of this study (Table 3) showed no cross-reactivity between caffeine and the test strips at 100,000 ng/mL suggesting a concentration dependent effect. Analysis of a series of caffeine concentrations (25 µg/mL to 10 mg/mL) demonstrated that the cross reactivity begins to occur at approximately 300,000 ng/mL (300 µg/mL) as shown in Fig. 4 and a false positive test result (complete absence of a test strip line) occurred between 7 and 10 mg/mL.

**Fig. 5:**
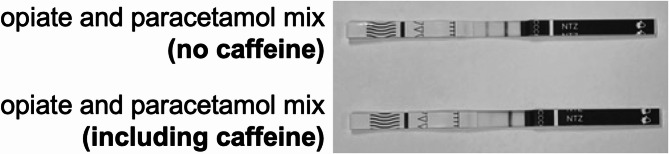
Images of testing strips following the analysis of an opiate/paracetamol mix with and without caffeine. The solution containing caffeine displays a faded test strip line

A solution was prepared to mimic seized samples, containing opiates and paracetamol, with and without caffeine. Each solution included 200 µg/mL of diamorphine, 6-MAM, 6-acetylcodeine, papaverine, noscapine, and paracetamol in tap water. One of these solutions also contained 300 µg/mL of caffeine. As shown in Fig. 5, no cross-reactivity was observed in the solution without caffeine, however the solution containing caffeine exhibited cross-reactivity with the test strip displaying a faded line.

Semi-quantitative analysis of the seized heroin samples, using a one-point calibration, determined an estimated caffeine content of 21.9–24.7%. Full results of this experimental work can be found in Table 4 and full analytical details relating to the semi-quantitative analysis can be found in Supplementary 4.

**Table 4:** Estimated caffeine content of seized samples and estimated concentration of caffeine in nitazene test strip solution

Seized sample	Estimated caffeine content (%)	Accurate mass of seized powder weighed into 1 mg/mL test strip solution (mg)	Estimated caffeine concentration in 1 mg/mL test strip solution (µg/mL)
A	24.7	1.3	321
B	21.9	1.2	262
C	23.2	1.3	301

The data presented in Table 4 corroborates the results depicted in Figs. 3 and 4, providing confirmation of the hypothesis that caffeine begins to cross-react with the strips at concentrations of approximately 300 µg/mL. This study highlights a significant challenge for harm reduction services, as caffeine is one of the most commonly detected cutting agents in illicit drug samples, including street heroin, cocaine, and amphetamines [19].

## Discussion

Nitazene test strips are currently being distributed by harm reduction services within the United Kingdom and used to identify the presence of nitazenes in seized, confiscated and surrendered drug samples. BTNX clearly state on their website that “*a positive or negative test result is NOT an indication that the substance being examined is safe to use*” and acknowledge that there “*is a possibility that technical or procedural errors as well as other substances and factors may interfere with the Rapid Response™ Nitazene Test Strip (Liquid / Powder) and cause false results*”. Despite these limitations, many organizations believe that drug testing strips remain a valuable harm reduction tool, not only for screening substances but also for facilitating conversations between harm reduction professionals and individuals about safer drug use practices.

Immunoassay test strips are typically simple to use and interpret, enabling individuals with no scientific background to independently use them. This removes the reliance of individuals on qualified scientific professionals, increasing their understanding of compounds present in their local market and ultimately leading to more informed drug use. Furthermore, when drug testing strips are distributed to individuals with a known history of drug use, they often share them with others. This facilitates a broader reach of these tools, fostering conversations within a traditionally hard-to-reach demographic about the risks associated with drug use.

However, care needs to be taken that the limitations of these test strips are clearly communicated. The results of this study demonstrate that it is possible that the presence of some nitazenes will not provide a positive result. This is particularly relevant to compounds such a metodesnitazene, detected in one death reported in England by the Office of Health Improvement and Disparities (OHID) in 2024 [20], and etazene, detected by WEDINOS in 2021 [9]. The detection of these compounds confirms that some nitazenes, undetected by test strips, are present in the UK drug supply. Harm reduction efforts must account for this risk, as false negatives could erode trust in these services and cause more harm.

In addition to the potential for false negatives, wide variation was observed in the LOD for each nitazene, ranging from 250 to 100,000 ng/mL. The manufactures of the test strips, BTNX, report the LOD of isotonitazene, protonitazene and *n*-pyrrolidinoetonitazne as 2000, 3000 and 1300 ng/mL respectively and Sisco et al. [21] reported the LOD for metonitazene as 1000 ng/mL. Additionally, De Vrieze et al. [22] noted a difference between test strip batches with one lot number providing an LOD for isotonitazene of 2000 ng/mL and the other 3000 ng/mL. The values reported within this study are consistent with those previously reported.

**Fig. 6:**
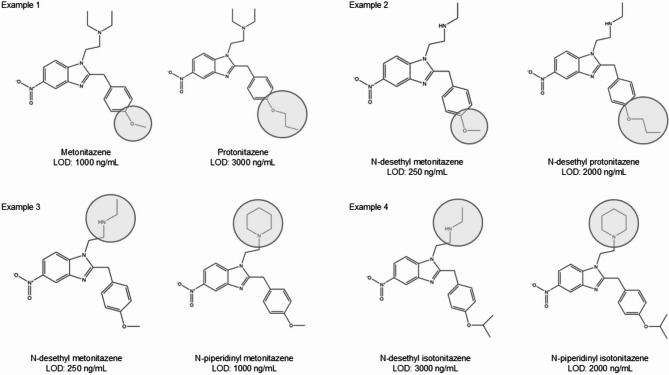
Examples of structural differences in nitazene analogs and the effects on LOD

There appears to be some structural explanation for the variation in the LOD observed between analogs. Examples of these structural changes are provided in Fig. 6 and the chemical structures of all nitazene analogs discussed in this paper can be found in Supplementary 2. For example, lengthening of the alkyloxy group appears to increase the LOD, such as between metonitazene and protonitazene with a LOD of 1000 ng/mL and 3000 ng/mL (Example 1, Fig. 6). The same is also observed for the *N*-desethyl-, *N*-pyrrolidino- and *N*-piperidinyl- analogs of these nitazenes, for example, *N*-desethyl metonitazene and *N*-desethyl protonitazene which have a LOD of 250 ng/mL and 2000 ng/mL respectively (Example 2, Fig. 6). However, this cannot be said for other substitutions which appear to have inconsistencies in regards to the effect of the substitution on the LOD, for example replacement of the *N*-desethyl group of *N*-desethyl metonitazene with a piperidinyl group, to form *N*-piperidinyl metonitazene, increases the LOD from 250 to 1000 ng/mL respectively (Example 3, Fig. 6); but conversely the same structural change does not have the same impact on *N*-desethyl isotonitazene and *N*-piperidinyl isotonitazene, 3000 ng/mL and 2000 ng/mL respectively (Example 4, Fig. 6). Therefore, it is recommended that no assumptions be made about the LOD of emerging nitazene analogs based solely on chemical structure. This study highlights the need to test newly emerging nitazene analogs using these test strips to ensure their on-going effectiveness.

Overall, the LOD and cross-reactivity results observed in this study were largely consistent with those reported by De Vrieze et al. [22] However, some differences were observed. Notably, 5-methyl etazene did not yield a positive result in our study at concentrations of 100 µg/mL, whereas De Vrieze et al. [22] reported cross-reactivity at 3 µg/mL. These discrepancies may be attributed to differences in sample preparation or potential variability between test strip batches. As previously discussed, De Vrieze et al. [22] also noted in their study variations in limits of detection between batches of test strips, which may have contributed to the observed differences. Importantly, this study corroborates their findings that removal of the 5-nitro group, to form ‘desnitazenes’ (e.g. metodesnitazene) prevents the detection of these analogs by the test strips.

Due to the high potency of nitazenes, the concentrations detected in drug samples are often significantly lower than those of other drugs of abuse. This is further complicated by the fact that individuals frequently engage in polydrug use, as well as frequent redosing. The presence of non-nitazene drugs and cutting agents was not found to interfere with the sensitivity of metonitazene demonstrating the ability of the test strips to detect nitazenes in isolation as well as in an adulterated drug sample.

De Vrieze et al. [22] examined the efficacy of the test strips using seized samples in Ghent. These samples composed of either metonitazene, protonitazene, isotonitazene, butonitazene, or *N*-pyrrolidino etonitazene with an estimated purity of > 90%. Although there is currently no data relating to the estimated purities of nitazene samples in the UK, it is understood that they are present as a minor constituent of street drug mixtures. As such the relatively high detection limits of the immunoassay test strips reported in this study, alongside the wide variation of those limits between analogs, may result in nitazenes going undetected, creating a false sense of safety for users, particularly when taking multiple doses or combining substances.

This study also highlights the risk of false positives due to high concentrations of caffeine, which causes significant fading of the test line from approximately 300 µg/mL. The high degree of fading when analyzing samples containing caffeine also resulted in the strips being more challenging to interpret. Previous studies have utilized image processing software such as ImageJ to aid interpretation of such results [22]. However, in a harm reduction setting, such an approach is unrealistic, and users must rely on their own interpretation which, in these examples, could easily lead to false conclusions that the samples contain nitazenes.

Diluting samples or adding less of a street sample to a solution could circumvent the issue of the observed caffeine cross reactivity. The manufacturers of the test strips, BTNX, now include small 1 mg spoons with each test strip, as a means of measuring the amount of street sample being added to the water, prior to testing. However, it is imperative to understand and consider that such an approach will also dilute the mass of nitazene present within the test solution. As previously mentioned, and shown in Table 2, the limits of detection for each nitazene can vary significantly. Therefore, diluting the sample carries an inherent risk of reducing the concentration of nitazenes below their detection threshold, leading to a false negative result.

As these strips cannot detect several nitazenes—some of which have been identified in the UK—have varying LODs and may produce false positives due to cross-reactivity with caffeine, alternative harm reduction strategies are needed. Many nitazenes are present at low concentration in the UK, and these strips risk producing false negatives which could provide individuals with a false sense of safety. This could significantly increase the risk of overdose or severe adverse reactions, especially in individuals who engage in repeated dosing.

## Limitations

Due to limited access to authentic seized samples containing nitazene compounds, the test strips could not be evaluated using such materials. Further investigation is required to determine the real-world applicability and performance of these test strips. This research was conducted using an available panel of drugs relative to the illicit drug market in the UK, therefore the cross-reactivity of all potential compounds has not been assessed. It is possible that there are other compounds which would benefit from assessment based on differing geographical locations. The strips were also assessed using nitazene compounds which have recently been controlled by the United Nations Office on Drugs and Crime and further assessment would need to be conducted in the likely event that additional nitazene compounds are detected in the global drug market.

## Conclusion

While immunoassay test strips can be valuable tools for harm reduction, their limitations must be carefully considered. This study highlights the challenges posed by false negative results, in particular in relation to nitazene analogs such as metodesnitazene which have been detected in post-mortem toxicology.

Furthermore, caffeine was shown to cross-react with the test strips at higher concentrations, providing a false positive result. The variation in the LOD for different nitazene analogs further complicates the use of these test strips, highlighting the need for continued testing to account for the detection of emerging substances. Complex street drug mixtures and polydrug use may contribute to inaccuracies in results, emphasizing the importance of clear communication about the limitations of immunoassay test strips to users.

Ultimately, while drug testing strips may play a role in harm reduction, this work highlights the need for their validation both in a laboratory setting and using authentic seized samples. This data is needed to ensure that any distribution of test strips is done after careful consideration of the limitations and risks involved, and that these are clearly communicated to service users.

## Supplementary Information

Below is the link to the electronic supplementary material.

Supplementary Material 1

## Data Availability

Data is provided within the manuscript or supplementary information files.

## Abbreviations

6-MAM: 6-Monoacetylmorphine
DRD: Drug-related deaths
LOD: Limit of detection
UK: United Kingdom
UNODC: United Nations Office on Drugs and Crime
USA: United States of America
WEDINOS: Welsh Emerging Drugs and Identification of Novel Substances
