# Supplementary material for: Evaluation of nitazene immunoassay test strips for rapid in-situ detection of nitazene and nitazene analogs in illicit drug samples
Source: Harm Reduct J. 2025 Aug 9;22:137. doi: 10.1186/s12954-025-01287-9 (PMC12335075; doi:10.1186/s12954-025-01287-9)
Supplement: Supplementary file 1 — Supplementary Material 1 [file 12954_2025_1287_MOESM1_ESM.docx]

**Supplementary 1 : Reference Standards**

Supplementary Table 1: Reference standards used in this study divided by drug class. Reference standards were obtained from LGC Standards (Middlesex, UK) Cayman Chemicals (Ann Arbor, MI, USA), Chiron AS (Trondheim, Norway), Lipomed AG (Arlesheim, Switzerland), MP Biomedicals (Santa Ana, CA, USA), Sigma Aldrich (Poole, UK), Thermo Scientific Chemicals (TCC) (UK), Tokyo Chemical Industry (TCI) Co Ltd (Tokyo, Japan) or VWR Chemicals (UK) or synthesized by the Sutcliffe Group at Manchester Metropolitan University (MMU), Manchester, UK ( > 98% purity) as described previously (Antonides et al. 2021^1^).

| **Drug Class** | **Compound** | **Formulation** | **Supplier** | **Purity** |
| --- | --- | --- | --- | --- |
| Non-Nitazene Opioids | Fentanyl HCl | Neat | MMU | >98% |
|  | Acetylfentanyl HCL | Neat | MMU | >98% |
|  | Acryloylfentanyl HCl | Neat | MMU | >98% |
|  | Benzoylfentanyl HCl | Neat | MMU | >98% |
|  | Butyrylfentayl HCl | Neat | MMU | >98% |
|  | Carfentanil HCl | 1000 𝜇g/mL in MeOH | Chiron | >97.8% |
|  | Crotonylfentanyl HCl | 1000 𝜇g/mL in MeOH | Chiron | >97.8% |
|  | Cyclopropylfentanyl | 1000 𝜇g/mL in MeOH | Chiron | >97.8% |
|  | 4-fluorobutyrylfentanyl HCl | 1000 𝜇g/mL in MeOH | Chiron | >97.8% |
|  | 2-Fluorofentanyl (o-fluorofentanyl) HCl | 1000 𝜇g/mL in MeOH | Chiron | >97.8% |
|  | 4-Fluorofentanyl (p-fluorofentanyl) HCl | 1000 𝜇g/mL in MeOH | Chiron | >97.8% |
|  | 4-fluoroisobutyrylfentanyl HCl | 1000 𝜇g/mL in MeOH | Chiron | >97.8% |
|  | Furanylfentanyl HCl | 1000 𝜇g/mL in MeOH | Chiron | >97.8% |
|  | Isovalerylfentanyl HCl | Neat | MMU | >98% |
|  | Methoxyacetylfentanyl HCl | 1000 𝜇g/mL in MeOH | Chiron | >97.8% |
|  | Norfentanyl | 1000 𝜇g/mL in MeOH | Chiron | >97.8% |
|  | Ocfentanil HCl | 1000 𝜇g/mL in MeOH | Chiron | >97.8% |
|  | Tetrahydrofuranylfentanyl (THFF) HCl | Neat | MMU | >98% |
|  | Valerylfentanyl HCl | Neat | MMU | >98% |
|  | Buprenorphine | 1000 𝜇g/mL in MeOH | Chiron | >97.8% |
|  | Brorphine | 1000 𝜇g/mL in MeOH | Chiron | >97.8% |
|  | Codeine HCl | Neat | Lipomed | >97% |
|  | Diamorphine | Neat | Sigma | >98% |
|  | Methadone Hydrochloride | Neat | Sigma | >98% |
|  | 6-Acetylcodeine Hydrochloride | Neat | Lipomed | >97% |
|  | 6-Monoacetyl morphine | Neat | Cayman | >98% |
|  | Noscapine | Neat | Sigma | 97% |
|  | Papaverine Hydrochloride | Neat | TCC | 99% |

| **Drug Class** | **Compound** | **Formulation** | **Supplier** | **Purity** |
| --- | --- | --- | --- | --- |
| Nitazene Analogs | Isotonitazene | 1000 𝜇g/mL in ACN | Chiron | >97.8% |
|  | Metonitazene HCl | Neat | Chiron | 97.5% |
|  | Protonitazene HCl | 1000 𝜇g/mL in MeOH | Chiron | >97.8% |
|  | Metodesnitazene HCl | 1000 𝜇g/mL in MeOH | Chiron | >97.8% |
|  | N-pyrrolidinoetonitazene | 1000 𝜇g/mL in MeOH | Chiron | >97.8% |
|  | N-desethylisonitazene HCl | 1000 𝜇g/mL in MeOH | Chiron | >97.8% |
|  | Etazene HCl | 1000 𝜇g/mL in MeOH | Chiron | >97.8% |
|  | Butonitazene | 1000 𝜇g/mL in MeOH | Chiron | >97.8% |
|  | Fluonitazene HCl | 1000 𝜇g/mL in MeOH | Chiron | >97.8% |
|  | 3-methoxymetodesnitazene citrate | Neat | Cayman | >98% |
|  | Protodesnitazene | Neat | Cayman | >98% |
|  | n-pyrrolidinometonitazene citrate | Neat | Cayman | >98% |
|  | 4-hydroxynitazene | Neat | Cayman | >98% |
|  | n-piperidinyl metonitazene citrate | Neat | Cayman | >98% |
|  | menitazene citrate | Neat | Cayman | >98% |
|  | n-pyrrolidinoisotonitazene citrate | Neat | Cayman | >98% |
|  | n-desethylmetonitazane hydrochloride | Neat | Cayman | >98% |
|  | n-piperidinyl 4'-hydroxynitazene citrate | Neat | Cayman | >98% |
|  | n-pyrrolidino metodesnitazene citrate | Neat | Cayman | >98% |
|  | n-desethylprotonitazene hydrochloride | Neat | Cayman | >98% |
|  | 5-methyl etazene citrate | Neat | Cayman | >98% |
|  | n-pyrrolidino 4'-hydroxynitazene citrate | Neat | Cayman | >98% |
|  | n-piperidinyl isotonitazene citrate | Neat | Cayman | >98% |
|  | n-pyrrolidinofluetonitazene citrate | Neat | Cayman | >98% |
|  | n-piperidinyl protonitazene citrate | Neat | Cayman | >98% |
|  | methylenedioxynitazene citrate | Neat | Cayman | >98% |
|  | ethyleneoxynitazene citrate | Neat | Cayman | >98% |
|  | 5-methylmetodesnitazene citrate | Neat | Cayman | >98% |
|  | sec-butonitazene | Neat | Cayman | >98% |
|  | n-desethyletonitazene | Neat | Cayman | >98% |
|  | Ethylene etonitazene citrate | Neat | Cayman | >98% |

| **Drug Class** | **Compound** | **Formulation** | **Supplier** | **Purity** |
| --- | --- | --- | --- | --- |
| Nitazene Analogs | Fluetonitazene citrate | Neat | Cayman | >98% |
|  | Propylnitazene citrate | Neat | Cayman | >98% |
|  | Clonitazene | Neat | Cayman | >98% |
|  | Etonitazene | Neat | Cayman | >98% |
|  | 5-aminoisotonitazene | 1000 𝜇g in 100 𝜇L ACN | Cayman | >98% |
| Synthetic Cannabinoids | 4F-ABUTINACA | Neat | Chiron | 99.9% |
|  | 4F-MDMB-BUTICA | Neat | Cayman | >98% |
|  | 5F-3,5-AB-PFUPPYCA | Neat | Chiron | 98.2% |
|  | 5F-ADB-PINACA | Neat | Chiron | 97.8% |
|  | 5F-BZO-POXIZID | Neat | Chiron | 99.9% |
|  | 5F-EMB-PICA | Neat | Cayman | >98% |
|  | 5F-MDMB-PICA | Neat | Chiron | 99.9% |
|  | 5F-MDA-19 | Neat | MMU | >98% |
|  | 5F-MPP-PICA | Neat | Cayman | >98% |
|  | 5F-PB-22 | 1000 𝜇g/mL in ACN | Chiron | >97.8% |
|  | AB-CHMINACA | Neat | MMU | >98% |
|  | ADB-4en-PINACA | Neat | MMU | >98% |
|  | ADB-5'Br-BUTINACA | Neat | Chiron | 98.9% |
|  | ADB-5'Br-INACA | Neat | Chiron | 99.8% |
|  | ADB-BUTINACA | Neat | MMU | >98% |
|  | ADB-FUBIATA | Neat | Chiron | 99.4% |
|  | ADB-HEXINACA | Neat | MMU | >98% |
|  | AMB-CHMICA | Neat | Chiron | 95.9% |
|  | AMB-FUBINACA | Neat | MMU | >98% |
|  | BZO-4en-POXIZID | Neat | Chiron | 96.7% |
|  | BZO-HEXOXIZID (MDA-19) | Neat | Chiron | 97.4% |
|  | CH-PIATA | Neat | Chiron | 97.5% |
|  | Cumyl-4CN-BINACA | 1000 𝜇g/mL in MeOH | Chiron | >97.8% |
|  | MDMB-4en-PINACA | Neat | MMU | >98% |
|  | MDMB-5'Br-INACA | Neat | Chiron | 99.6% |
|  | MDMB-B(enz)ICA | Neat | MMU | >98% |
|  | MDMB-CHMICA | Neat | MMU | >98% |
|  | PB-22 | 1000 𝜇g/mL in ACN | Chiron | >97.8% |
|  | MDMB-INACA | Neat | Cayman | >98% |
| Benzodiazepine-type substances | Alprazolam | 1000 𝜇g/mL in MeOH | Chiron | >97.8% |
|  | Bromazolam | 1000 𝜇g/mL in MeOH | Chiron | >97.8% |
|  | Clonazepam | Neat | Sigma | >98% |
|  | Clonazolam | Neat | Chiron | 98.5% |
|  | Clobromazolam | Neat | Chiron | 99.9% |

| **Drug Class** | **Compound** | **Formulation** | **Supplier** | **Purity** |
| --- | --- | --- | --- | --- |
| Benzodiazepine-type substances | Desalkylgidazepam | Neat | Chiron | 98.8% |
|  | Diazepam | Neat | Sigma | >98% |
|  | Diclazepam | Neat | Chiron | 99.0% |
|  | Etizolam | Neat | Chiron | 99.4% |
|  | Flualprazolam | Neat | Chiron | 99.4% |
|  | Flubromazepam | Neat | Chiron | 99.6% |
|  | Flubromazolam | Neat | Chiron | 99.1% |
|  | Midazolam | 1000 𝜇g/mL in MeOH | Chiron | >97.8% |
|  | Nitrazepam | Neat | Sigma | >98% |
|  | Phenazepam | Neat | Sigma | 99.3% |
| Cutting Agents and Adulterants | Benzocaine | Neat | Sigma | >99% |
|  | Caffeine anhydrous | Neat | Sigma | >99% |
|  | Diphenhydramine HCl | Neat | Sigma | >98% |
|  | Levamisole HCl | Neat | Sigma | >98% |
|  | Lidocaine HCl | Neat | Sigma | >99.8% |
|  | Paracetamol (Acetominophen) | Neat | Sigma | >98% |
|  | Phenacetin | Neat | VWR | >99% |
|  | Glucose | Neat | Sigma | >99.5% |
|  | Magnesium Sulphate | Neat | VWR | >99% |
|  | Sodium bicarbonate | Neat | Sigma | >99% |
|  | Citric acid (monohydrate) | Neat | TCC | >99% |
| Steroids | Methylandrostanolone | Neat | TCI | 98.8% |
|  | Stanozolol | Neat | LGC | 99.52% |
|  | Methyltestosterone | Neat | Sigma | 99% |
|  | Oxymetholone | Neat | Chiron | 98% |
|  | Methandienone | Neat | TCI | 97.5% |
| Other | Cocaine HCl | Neat | Sigma | >98% |
|  | Cocaine (Base) | 1mg/mL in ACN | Sigma | >99% |
|  | Gabapentin | Neat | Sigma | >98% |
|  | Ketamine HCl | Neat | Sigma | >98% |
|  | MDMA HCl | Neat | Sigma | >98% |
|  | Methamphetamine HCl | Neat | Sigma | >98% |
|  | Pregabalin | Neat | Lipomed | >97% |
|  | Delta-9-THC | Neat | Lipomed | >97% |
|  | Dimethylpentylone | Neat | Chiron | 98.2 % |

1. Antonides LH, Cannaert A, Norman C, NicDáeid N, Sutcliffe OB, Stove CP, et al. Shape matters: The application of activity-based in vitro bioassays and chiral profiling to the pharmacological evaluation of synthetic cannabinoid receptor agonists in drug-infused papers seized in prisons. Drug Testing and Analysis. 2021;13(3):628-43.

**Supplementary 2: Nitazene Analog Chemical Structures**

Supplementary Table 3: Chemical structures of nitazene analogs used in this study.

| 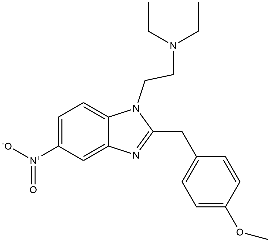 | 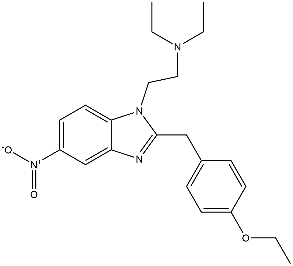 | 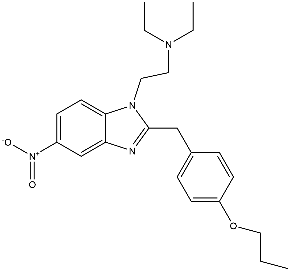 | 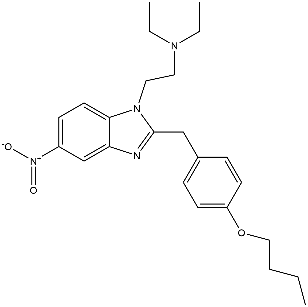 |
| --- | --- | --- | --- |
| Metonitazene  Test Strip Result: **Positive** | Etonitazene  Test Strip Result: **Positive** | Protonitazene  Test Strip Result: **Positive** | Butonitazene  Test Strip Result: **Positive** |
| 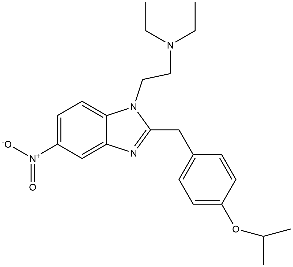 | 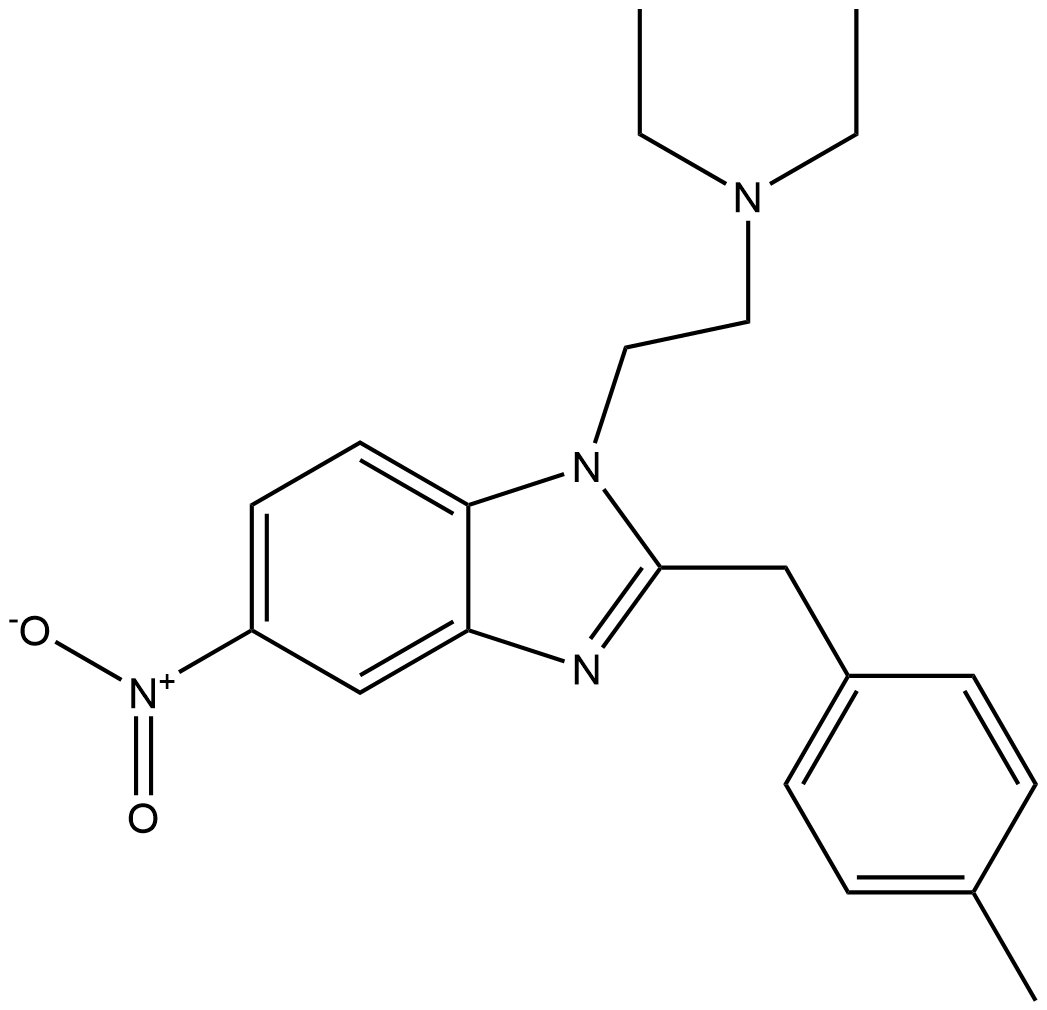 | 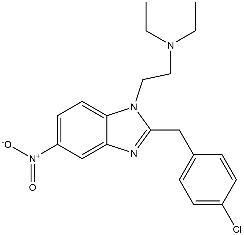 | 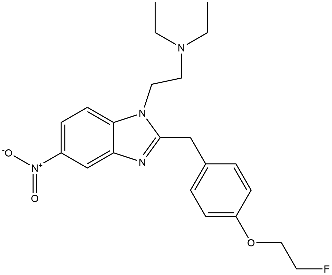 |
| Isotonitazene  Test Strip Result: **Positive** | Menitazene  Test Strip Result: **Positive** | Clonitazene  Test Strip Result: **Positive** | Flunitazene  Test Strip Result: **Positive** |
| 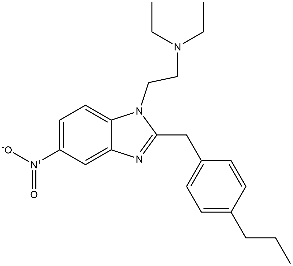 | 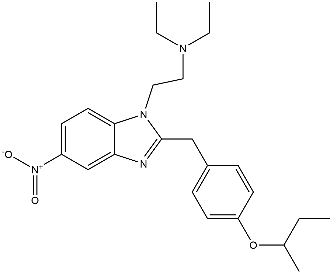 | 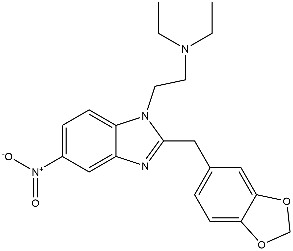 | 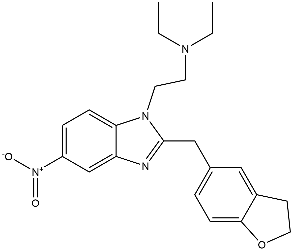 |
| Propylnitazene  Test Strip Result: **Positive** | *Sec*- butonitazene  Test Strip Result: **Positive** | Methylenedioxy nitazene  Test Strip Result: **Positive** | Ethylene oxynitazane  Test Strip Result: **Positive** |
| 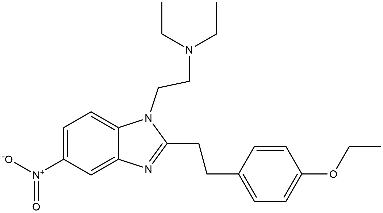 | 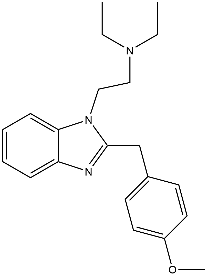 | 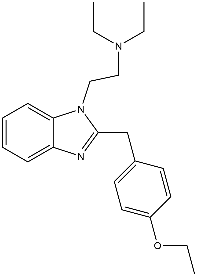 | 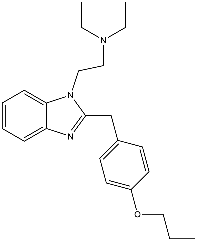 |
| Ethylene etonitazene  Test Strip Result: **Negative** | Metodesnitazene  Test Strip Result: **Negative** | Etazene  Test Strip Result: **Negative** | Protodesnitazene  Test Strip Result: **Negative** |
| 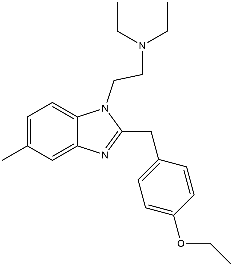 | 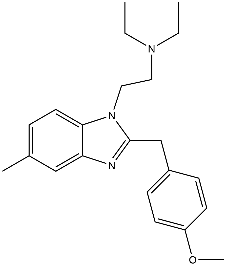 | 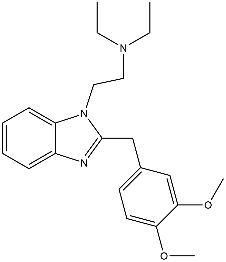 | 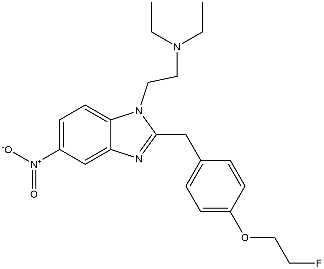 |
| 5-methyl etazene  Test Strip Result: **Negative** | 5-methyl metodesnitazene  Test Strip Result: **Positive** | 3-methoxy metodesnitazene  Test Strip Result: **Negative** | Fluetonitazene  Test Strip Result: **Positive** |
| 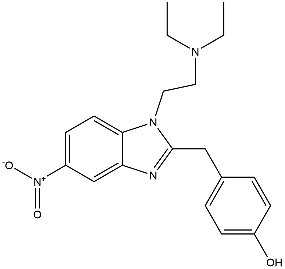 | 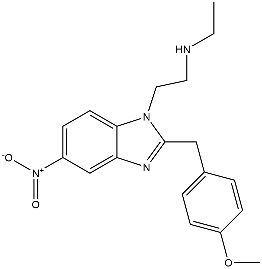 | 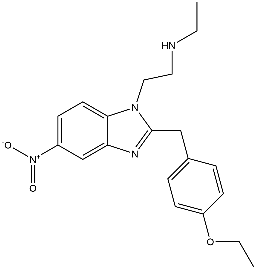 | 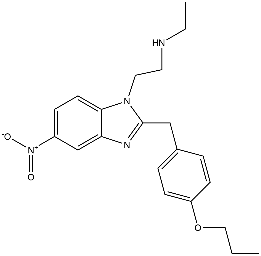 |
| 4’-hydroxy nitazene  Test Strip Result: **Positive** | n-desethyl metonitazene  Test Strip Result: **Positive** | n-desethyl etonitazene  Test Strip Result: **Positive** | n-desethyl protonitazene  Test Strip Result: **Positive** |
| 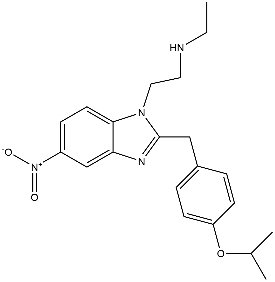 | 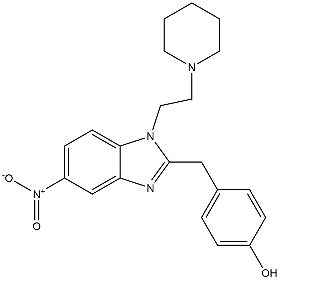 | 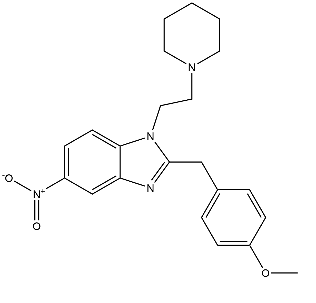 | 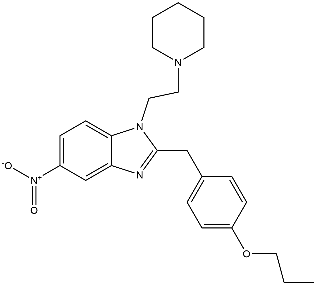 |
| n-desethyl isotonitazene  Test Strip Result: **Positive** | n-piperidinyl 4'-hydroxynitazene  Test Strip Result: **Positive** | n-piperidinyl metonitazene  Test Strip Result: **Positive** | n-piperidinyl protonitazene  Test Strip Result: **Positive** |
| 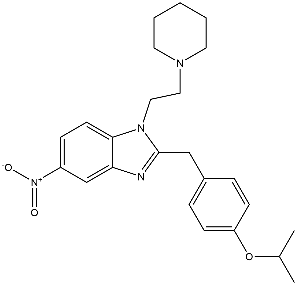 | 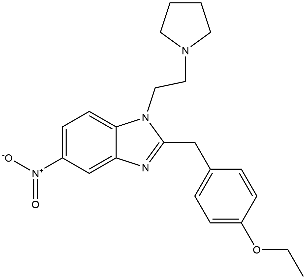 | 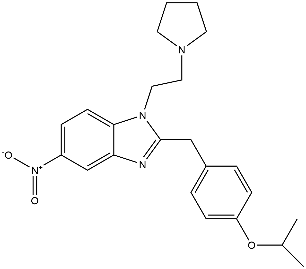 | 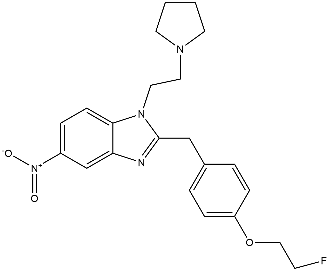 |
| n-piperidinyl isotonitazene  Test Strip Result: **Positive** | N-pyrrolidino etonitazene  Test Strip Result: **Positive** | N-pyrrolidino isotonitazene  Test Strip Result: **Positive** | n-pyrrolidino fluetonitazene  Test Strip Result: **Positive** |
| 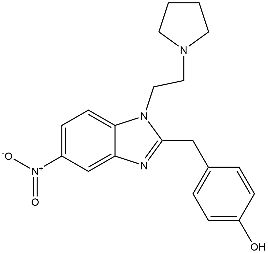 | 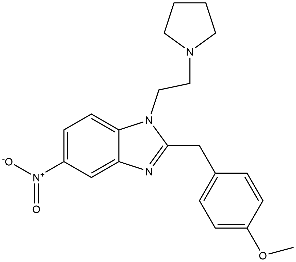 | 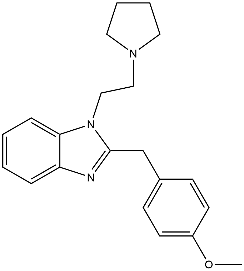 | 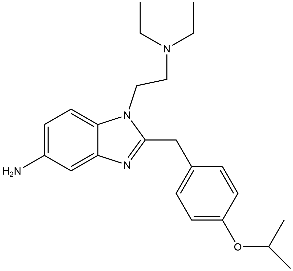 |
| n-pyrrolidino 4'-hydroxy nitazene  Test Strip Result: **Positive** | n-pyrrolidino metonitazene  Test Strip Result: **Positive** | n-pyrrolidino metodesnitazene  Test Strip Result: **Negative** | 5-aminoisotonitazene  Test Strip Result: **Negative** |

**Supplementary 3: Cross Reactivity Testing**

Supplementary Table 3: Results of cross reactivity testing.

| **Compound Class** | **Compound (100 ug/mL)** | **Test Strip Result** |
| --- | --- | --- |
| Non-Nitazene Opioids | 2-fluorofentanyl (o-fluorofentanyl) HCl | Negative |
|  | 4-fluorobutyrylfentanyl HCl | Negative |
|  | 4-fluorofentanyl (p-fluorofentanyl) HCl | Negative |
|  | 4-fluoroisobutyrylfentanyl HCl | Negative |
|  | 6-monoacetylmorphine | Negative |
|  | Acetylfentanyl HCL | Negative |
|  | Acryloylfentanyl HCl | Negative |
|  | Benzoylfentanyl HCl | Negative |
|  | Brorphine | Negative |
|  | Buprenorphine | Negative |
|  | Butyrylfentayl HCl | Negative |
|  | Carfentanil HCl | Negative |
|  | Codeine HCl | Negative |
|  | Crotonylfentanyl HCl | Negative |
|  | Cyclopropylfentanyl | Negative |
|  | Diamorphine | Negative |
|  | Fentanyl HCl | Negative |
|  | Furanylfentanyl HCl | Negative |
|  | Isovalerylfentanyl HCl | Negative |
|  | Methadone | Negative |
|  | Methoxyacetylfentanyl HCl | Negative |
|  | Norfentanyl | Negative |
|  | Ocfentanil HCl | Negative |
|  | Tetrahydrofuranylfentanyl (THFF) HCl | Negative |
|  | Valerylfentanyl HCl | Negative |
| Synthetic Cannabinoids | 4F-ABUTINACA | Negative |
|  | 4F-MDMB-BUTICA | Negative |
|  | 5F-3,5-AB-PFUPPYCA | Negative |
|  | 5F-ADB-PINACA | Negative |
|  | 5F-BZO-POXIZID | Negative |
|  | 5F-EMB-PICA | Negative |
|  | 5F-MDA-19 | Negative |
|  | 5F-MDMB-PICA | Negative |
|  | 5F-MPP-PICA | Negative |
|  | 5F-PB-22 | Negative |
|  | AB-CHMINACA | Negative |
| **Compound Class** | **Compound (100 ug/mL)** | **Test Strip Result** |
| Synthetic Cannabinoids | ADB-4en-PINACA | Negative |
|  | ADB-5'Br-BUTINACA | Negative |
|  | ADB-5'Br-INACA | Negative |
|  | ADB-BUTINACA | Negative |
|  | ADB-FUBIATA | Negative |
|  | ADB-HEXINACA | Negative |
|  | AMB-CHMICA | Negative |
|  | AMB-FUBINACA | Negative |
|  | BZO-4en-POXIZID | Negative |
|  | BZO-HEXOXIZID (MDA-19) | Negative |
|  | CH-PIATA | Negative |
|  | Cumyl-4CN-BINACA | Negative |
|  | MDMB-4en-PINACA | Negative |
|  | MDMB-5'Br-INACA | Negative |
|  | MDMB-B(enz)ICA | Negative |
|  | MDMB-CHMICA | Negative |
|  | MDMB-INACA | Negative |
|  | PB-22 | Negative |
| Benzodiazepine-type substances | Alprazolam | Negative |
|  | Bromazolam | Negative |
|  | Clobromazolam | Negative |
|  | Clonazepam | Negative |
|  | Clonazolam | Negative |
|  | Desalkylgidazepam | Negative |
|  | Diazepam | Negative |
|  | Diclazepam | Negative |
|  | Etizolam | Negative |
|  | Flualprazolam | Negative |
|  | Flubromazepam | Negative |
|  | Flubromazolam | Negative |
|  | Midazolam | Negative |
|  | Nitrazepam | Negative |
|  | Phenazepam | Negative |
| Cutting Agents and Adulterants | Benzocaine | Negative |
|  | Caffeine | Negative |
|  | Citric acid | Negative |
|  | Diphenhydramine HCl | Negative |
|  | Glucose | Negative |
|  | Levamisole HCl | Negative |

| **Compound Class** | **Compound (100 ug/mL)** | **Test Strip Result** |
| --- | --- | --- |
| Cutting Agents and Adulterants | Lidocaine HCl | Negative |
|  | Magnesium Sulphate | Negative |
|  | Paracetamol | Negative |
|  | Phenacetin | Negative |
|  | Sodium bicarbonate | Negative |
| Steroids | Methylandrostanolone | Negative |
|  | Methandienone | Negative |
|  | Methyltestosterone | Negative |
|  | Oxymetholone | Negative |
|  | Stanozolol | Negative |
| Other | Cocaine (Base) | Negative |
|  | Cocaine HCl | Negative |
|  | Delta-9-THC | Negative |
|  | Dimethylpentylone | Negative |
|  | Gabapentin | Negative |
|  | Ketamine HCl | Negative |
|  | MDMA HCl | Negative |
|  | Methamphetamine HCl | Negative |
|  | Pregabalin | Negative |

**Supplementary 4: GC-MS Analysis of Seized Samples**

**Methods and Materials**

**Materials**

LC-MS grade methanol was purchased from Fisher Scientific, UK; [bupivacaine](https://www.sciencedirect.com/topics/pharmacology-toxicology-and-pharmaceutical-science/bupivacaine) was obtained from Sigma-Aldrich (Poole, UK).

**Reference Standards**

All reference standards used are listed above in Supplementary 1.

**Methods**

**Extraction of Seized Samples**

All samples were powders. Approximately 5 mg of the powders were extracted in 1 mL of 0.25 mg/mL bupivacaine (internal standard) in methanol and sonicated (5 min) and the supernatants were qualitatively analyzed by GC-MS.

**Preparation of Caffeine Reference Standard (1mg/mL)**

A 1 mg/mL caffeine reference standard was prepared by weighing 5 mg of caffeine reference standard into a 5 mL volumetric flask on a calibrated balance. 5 mL of 0.25 mg/mL bupivacaine (internal standard) in methanol was added to the flask and the solution was transferred to a vial and sealed immediately.

**Instrumental Analysis**

GC-MS analysis of sample extracts was performed using a 7820 A gas chromatograph coupled to a 5977E mass spectrometer (Agilent technologies, Santa Clara, CA, USA). Injection mode: 1 μL sample injection was used with a 20:1 split into a 4 mm internal diameter deactivated glass liner pre-packed with quartz wool, injection port temperature: 200ºC, carrier gas: He, flow: 1 mL/min. Column: HP-5MS, 0.33 μm, 0.2 mm × 25 m (Agilent Technologies). GC oven: 80ºC held for 3 min; 40ºC/min to 300ºC held for 6 min; total run time: 14.5 min; transfer line: 295ºC. The mass spectrometer operated in [electron ionization](https://www.sciencedirect.com/topics/pharmacology-toxicology-and-pharmaceutical-science/electron-ionization) (EI) mode. Ionization conditions: 70 eV in full scan mode (50–550 amu), ion source: 230ºC, quadrupole: 150ºC.

Compound identification by GC-MS was carried out by comparison of the mass spectra in seized samples the SWGDRUG mass spectral library (version 3.12, released 16 January 2023). A reverse match (RMatch) factor, which measures the difference between the mass spectrum of the unknown chromatographic peak to spectra held in the spectra library, was required to be greater than 850/1,000 for positive identification.

**Calculation of Approximate Caffeine Concentration**

The caffeine concentration of each seized sample was estimated using a single point calibration (1 mg/mL). Each seized sample and the caffeine reference standard were analysed on the same day using the GC-MS method described above. The estimated caffeine concentration was subsequently calculated using Equation 1.

$$\frac{Peak Area of Reference Standard}{Peak Area of Unknown}= \frac{Concentration of Standard}{Concentration of Unknown}$$

Equation 1 : Calculation of approximate caffeine concentration using single point calibration

**Results**

**Caffeine 1mg/mL Reference Standard**


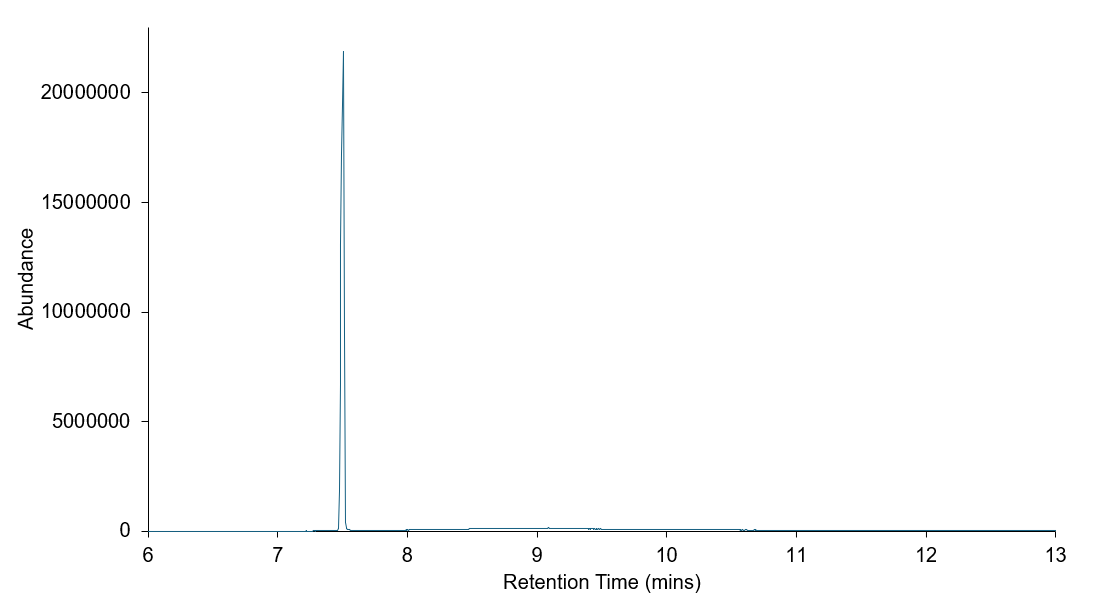


**Supplementary Figure 1: GC-MS Chromatogram, caffeine reference standard (1mg/mL)**

**Supplementary Table 5: Analytical data relating to caffeine reference standard (1mg/mL)**

| **Retention Time**  **(mins)** | **Identification** | **Reverse Match Factor (RMF)** | **Peak Area** |
| --- | --- | --- | --- |
| **7.508** | **Caffeine** | **961** | **383418011** |

**Seized Sample (A)**


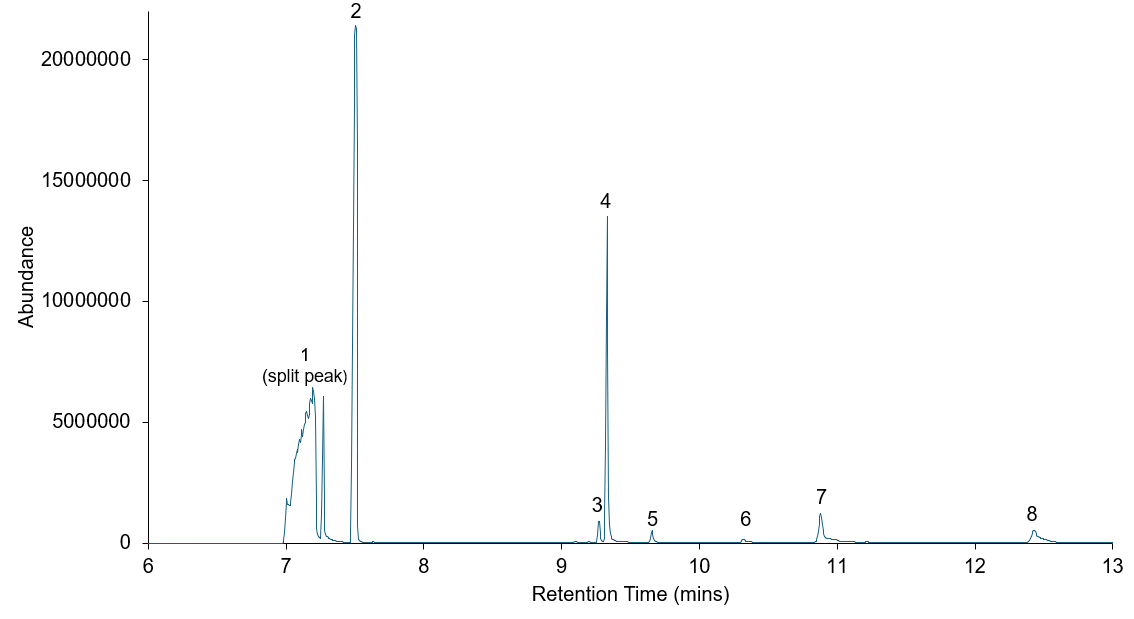


**Supplementary Figure 2: GC-MS Chromatogram, seized sample (a).**

**Supplementary Table 6: Analytical data relating to seized sample (a)**

| **Weight of powder: 4.5 mg in 1mL** | | | | | | |
| --- | --- | --- | --- | --- | --- | --- |
| **Peak Number** | **Retention Time**  **(mins)** | **Identification** | **Reverse Match Factor (RMF)** | **Peak Area** | **Estimated**  **Caffeine**  **Concentration (mg) mL** | **Estimated Caffeine Concentration**  **(%)** |
| **1** | **7.195** | **Paracetamol** | **959** | **607144614** |  |  |
| **2** | **7.507** | **Caffeine** | **963** | **424896006** | **1.11** | **24.7** |
| **3** | **9.272** | **6-Acetylcodeine** | **918** | **9118535** |  |  |
| **4** | **9.332** | **6-monoacetylmorphine** | **957** | **137913095** |  |  |
| **5** | **9.657** | **Diacetylmorphine** | **939** | **5823441** |  |  |
| **6** | **10.321** | **Papaverine** | **863** | **2725457** |  |  |
| **7** | **10.882** | **Sucrose Octaacetate** | **903** | **22473003** |  |  |
| **8** | **12.431** | **Noscapine** | **916** | **21506595** |  |  |

**Supplementary Table 7: Estimated Caffeine Concentration (µg/mL) in Test Strip Solution (seized sample (a)**

| **Weight of Seized Sample in 1 mL**  **(mg)** | **Estimated Caffeine Concentration**  **(As calculated from GC-MS)**  **(%)** | **Estimated Caffeine Concentration in Test Strip Solution**  **(µg/mL)** |
| --- | --- | --- |
| **1.3** | **24.7** | **321** |

**Seized Sample (B)**


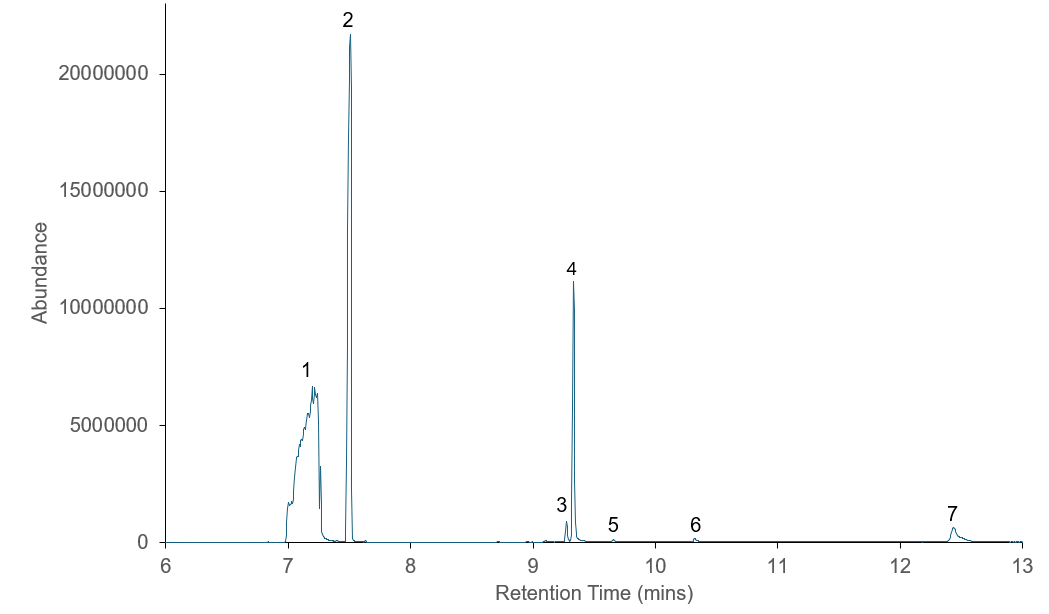


**Supplementary Figure 3: GC-MS Chromatogram, seized sample (b).**

**Supplementary Table 8: Analytical data relating to seized sample (b)**

| **Weight of powder: 5.2 mg** | | | | | | |
| --- | --- | --- | --- | --- | --- | --- |
| **Peak Number** | **Retention Time**  **(mins)** | **Identification** | **Reverse Match Factor (RMF)** | **Peak Area** | **Estimated**  **Caffeine**  **Concentration (mg/ mL)** | **Estimated Caffeine Concentration(%)** |
| **1** | **7.195** | **Paracetamol** | **960** | **699341909** |  |  |
| **2** | **7.510** | **Caffeine** | **960** | **437123278** | **1.14** | **21.9** |
| **3** | **9.276** | **6-Acetylcodeine** | **931** | **8542247** |  |  |
| **4** | **9.334** | **6-monoacetylmorphine** | **959** | **115380413** |  |  |
| **5** | **9.658** | **Diacetylmorphine** | **851** | **1049392** |  |  |
| **6** | **10.326** | **Papaverine** | **873** | **3007398** |  |  |
| **7** | **12.439** | **Noscapine** | **908** | **26992570** |  |  |

**Supplementary Table 9: Estimated Caffeine Concentration (µg/mL) in Test Strip Solution (seized sample (a)**

| **Weight of Seized Sample in 1 mL** | **Estimated Caffeine Concentration**  **(As calculated from GC-MS)**  **(%)** | **Estimated Caffeine Concentration in Test Strip Solution**  **(µg/mL)** |
| --- | --- | --- |
| **1.2** | **21.9** | **262** |

**Seized Sample (C)**


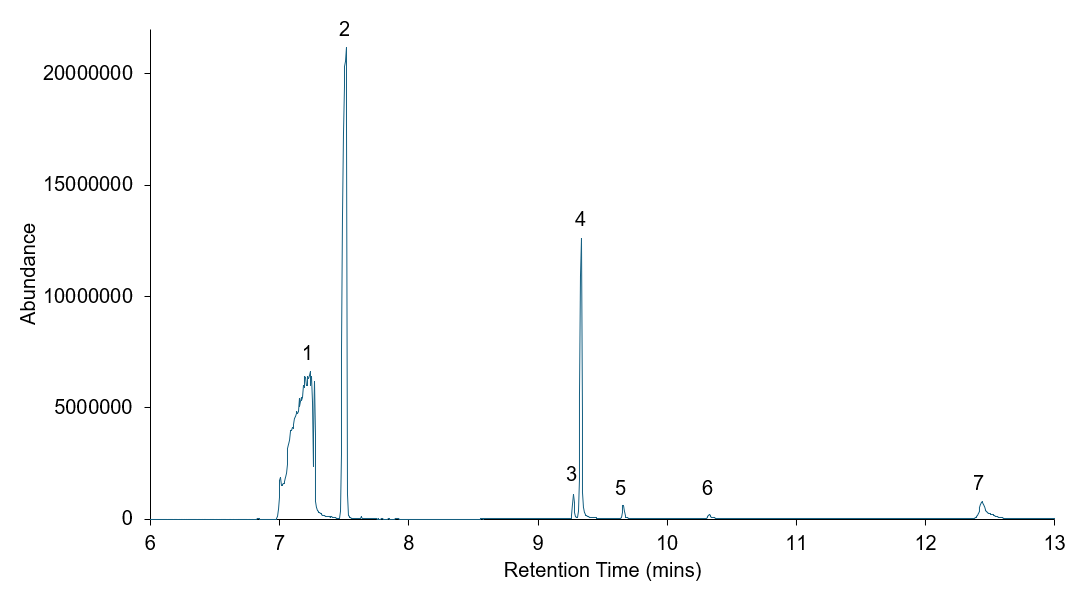


**Supplementary Figure 3: GC-MS Chromatogram, seized sample (c).**

**Supplementary Table 10: Analytical data relating to seized sample (c)**

| **Weight of powder: 5.5 mg** | | | | | | |
| --- | --- | --- | --- | --- | --- | --- |
| **Peak Number** | **Retention Time**  **(mins)** | **Identification** | **Reverse Match Factor (RMF)** | **Peak Area** | **Estimated**  **Caffeine**  **Concentration (mg/ mL)** | **Estimated Caffeine Concentration**  **(%)** |
| **1** | **7.235** | **Paracetamol** | **956** | **748766911** |  |  |
| **2** | **7.515** | **Caffeine** | **962** | **488739153** | **1.27** | **23.2** |
| **3** | **9.275** | **6-Acetylcodeine** | **902** | **10391872** |  |  |
| **4** | **9.336** | **6-monoacetylmorphine** | **957** | **133569428** |  |  |
| **5** | **9.661** | **Diacetylmorphine** | **943** | **7096682** |  |  |
| **6** | **10.326** | **Papaverine** | **880** | **3747467** |  |  |
| **7** | **12.438** | **Noscapine** | **915** | **33052419** |  |  |

**Supplementary Table 11: Estimated Caffeine Concentration (µg/mL) in Test Strip Solution (seized sample (a)**

| **Weight of Seized Sample in 1 mL** | **Estimated Caffeine Concentration**  **(As calculated from GC-MS)**  **(%)** | **Estimated Caffeine Concentration in Test Strip Solution**  **(µg/mL)** |
| --- | --- | --- |
| **1.3** | **23.2** | **301** |
